# Solid Tumor Differentiation Therapy – Is It Possible?

**DOI:** 10.18632/oncotarget.512

**Published:** 2012-05-24

**Authors:** Filemon Dela Cruz, Igor Matushansky

**Affiliations:** ^1^ Division of Pediatric Oncology, Department of Pediatrics, Columbia University College of Physicians and Surgeons, New York, NY; ^2^ Division of Medical Oncology, Department of Medicine, Columbia University College of Physicians and Surgeons, New York, NY

**Keywords:** Differentiation therapy, soft tissue sarcoma, retinoids, histone deacetylase inhibitor, PPAR-gamma agonist, trabectedin

## Abstract

Genetic and epigenetic events within a cell which promote a block in normal development or differentiation coupled with unregulated proliferation are hallmarks of neoplastic transformation. Differentiation therapy involves the use of agents with the ability to induce differentiation in cells that have lost this ability, i.e. cancer cells. The promise of differentiation-based therapy as a viable treatment modality is perhaps best characterized by the addition of retinoids in the treatment of acute promyelocytic leukemia (APML) revolutionizing the management of APML and dramatically improving survival. However, interest and application of differentiation-based therapy for the treatment of solid malignancies have lagged due to deficiencies in our understanding of differentiation pathways in solid malignancies. Over the past decade, a differentiation-based developmental model for solid tumors has emerged providing insights into the biology of various solid tumors as well as identification of targetable pathways capable of re-activating blocked terminal differentiation programs. Furthermore, a variety of agents including retinoids, histone deacetylase inhibitors (HDACI), PPARγ agonists, and others, currently in use for a variety of malignancies, have been shown to induce differentiation in solid tumors. Herein we discuss the relevancy of differentiation-based therapies in solid tumors, using soft tissue sarcomas (STS) as a biologic and clinical model, and review the preclinical data to support its role as a promising modality of therapy for the treatment of solid tumors.

## INTRODUCTION

Differentiation therapy is a therapeutic modality aimed at re-activating endogenous differentiation programs in cancer cells with subsequent tumor cellular maturation and concurrent loss of the tumor phenotype. In modern literature, one can trace the conceptual origins of differentiation therapy to the work of G. Barry Pierce who posited that malignant cells could differentiate into non-malignant cells [1, 2]. Although a theoretically attractive option, differentiation therapy has historically been greatly hampered by both our practical lack of understanding of the biology of normal differentiation pathways as well as by our theoretical inability to envision a methodology that could restore or supersede a tumor's immutable genetic level mutations which result in the lineage specific block to differentiation and subsequent tumorigenesis. Another long standing drawback of differentiation therapy has been its practical and theoretical disadvantage as compared to novel and even conventional cytotoxic approaches. Cytotoxic chemotherapy aims directly at cancer cell death while differentiation therapy alludes to a more complex and more nebulous process of cancer to normal tissue transitioning.

However, if there is one thing we have learned over the last 50 years of treating cancer patients, it is that conventional approaches (e.g., conventional cytotoxic agents, targeted antibodies or small molecule inhibitors) are not sufficient in effecting cures for a significant proportion of cancer patients. According to the American Cancer Society register, this year more than 500,000 Americans will die of cancer accounting for nearly 1 of every 4 deaths [3]. And, while the 5-year survival rate for all patients diagnosed with cancer in the last ten years is 66%, up from the 50% rate of the 1970s [3], that means, as of today, one out of every three patients diagnosed with cancer will not be alive five years later. Clearly there is reason to pursue all potential therapeutic options.

However, is differentiation therapy a “realistic” potential therapeutic option? Is it really possible to make cancer – “normal”? In the era before retinoic acid-based differentiation therapy for acute promyelocytic leukemia (APML), through the use of various cytotoxic chemotherapies the remission rates had progressively improved from 50 to 80%, of which only about 35% could expect to be long term survivals. However, now with the use of retinoic acid and chemotherapy more than 90% of patients with newly diagnosed APML patients can achieve complete remission and about 75% can be cured [4-13]. Mechanistically, APML cells in the vast majority of patients have a characteristic chromosomal translocation t(15;17) that produces the fusion gene consisting of the promyelocytic leukemia (PML) and retinoic acid receptor α (RARα) genes. PML-RARα retains critical domains of PML and RARα, and plays a key role in the pathogenesis of APML by recruiting transcriptional repressors, histone deacetylases (HDACs), and DNA methyltransferases. Pharmacological doses of ATRA trigger dissociation of PML-RARα/HDAC complexes resulting in degradation of PML-RARα and resumption of myeloid differentiation in APML cells [14]. Explained in the above manner – the use of retinoic acid to “relieve” the differentiation block makes complete sense.

So why aren't there more examples of differentiation therapy? For hematopoietic malignancies it has been argued that APML represents an isolated example of a “simple karyotype” disease that is both addicted to the characteristic fusion gene for tumorigenicity and has minimal other genetic abnormalities. Thus, the reversal of one pathway is sufficient to reverse the tumorigenicity of APML and place it back on the road to normal differentiation, something that is unlikely to be true for other hematopoietic malignancies. For solid tumors, the application of differentiation therapy has been further compounded by the absence of developmental models of cancer progression that correlate cancer subtypes to stages of normal development. Models such as those proposed by Pierce and colleagues have been historically minimized in favor of the more common notions of solid tumorigenesis which hypothesize that mutations occurring in “normal” tissue results in gradual dedifferentiation to cancer cells harboring features of normal tissue reflecting varying degrees of differentiation – a feature invariably linked to cancer aggressiveness [15]. Therefore, even the consideration of differentiation-based therapy in solid tumors has been hampered by the lack of developmental-based classifications of solid tumors.

In this review, we discuss how recent advances in the developmental-based classification of solid tumors are paving the way and leading to novel differentiation-based therapies for solid tumors.

## Differentiation-based classification models of cancer

Similar to other solid tumors, sarcomas have been historically classified based on histopathological features reflecting the degree to which these tumors resemble normal tissue. However, unlike other solid tumors, the existence of a connective tissue (mesenchymal) stem cell, along with *in vitro* methodologies to differentiate them into mature tissues, have allowed us, for the first time, to query whether sarcoma subtypes arise as a result of cellular transformation at discrete stages of differentiation [16]. Through gene clustering and distance correlation analyses, our group was able to correlate the expression signatures of each liposarcoma subtype to a corresponding point along the adipocytic differentiation time course providing evidence that the dedifferentiated and pleomorphic liposarcoma subtypes represent cells arrested at an early point in differentiation compared to myxoid/round-cell and well-differentiated cells which arrest at later and more mature stages of development. Furthermore, our analysis of differentially expressed genes identified genes marking discrete stages of adipocytic differentiation and discriminating these genes from markers that may be involved in malignant transformation and potentially amenable to therapeutic targeting. Picking up on this theme, and using significantly advanced computational methodologies, Riester and colleagues recently developed a statistical algorithm utilizing gene expression data from different cancers (including AML, breast carcinoma and liposarcoma) to construct phylogenetic trees which objectively and systematically categorized cancer subtypes based on degrees of maturation and relative to their corresponding cells of origin (e.g. hMSC for liposarcomas) [17]. The algorithm proposed successfully classified: (1) the AML subtypes in accord with the FAB classification schema (e.g. M0 subtype was arrayed closest to stem cells); (2) breast carcinoma based on estrogen receptor (ER) status; and (3) confirmed our initial findings in liposarcomas as described above. This developmental-based approach represents not only a new method for reclassifying solid tumors, but also provides fundamental insight into solid tumor etiology.

## Targeting of differentiation pathways

Along with the changing classification systems that now plot solid tumors onto developmental maps, we are getting better at understanding how to activate differentiation pathways in cancers so as to progress them along their developmental paths. Using this rationale, we have previously shown that mesenchymal stem cells (MSCs) are the progenitors of malignant fibrous histiocytoma (MFH; now termed high grade undifferentiated pleomorphic sarcoma [HGUPS], a commonly diagnosed mesenchymal tumor) and that increased levels of DKK1, a Wnt developmental pathway inhibitor, mediate the transition from the MSC state to the MFH state [18]. Perhaps, more importantly, we have been able to demonstrate that MFH cells in which Wnt signaling is re-established to mirror the MSC-state become amenable to differentiation into mature connective tissue lineages with concurrent loss of tumor cell properties [18]. Although a novel finding at the time, if one looks closely enough, there are many agents already in clinical practice that may function as differentiation agents.

## Histone deacetylase inhibitors

Epigenetic modifications which affect the chromatin architecture have been implicated in malignant progression and transformation [19]. Histone deacetylation, mediated by histone deacetylases (HDACs), leading to chromatin compaction is associated with transcriptional repression of tumor suppressors involved in regulating cell growth and differentiation in different cancers including sarcomas [20, 21]. Hence, there has been considerable interest in HDAC inhibitors (HDACIs) and preclinical data to suggest a differentiation indcuing effect of HDACIs in a variety of solid tumor and sarcoma models [22-26].

Platta and colleagues showed that a small cell lung carcinoma cell line, DMS53, underwent dramatic morphological changes suggestive of cellular differentiation following treatment with the histone deacetylase inihibitor (HDACI), trichostatin A [27]. Rephaeli and colleagues showed that treatment of mice with established 22Rv1 prostate tumors with AN-7, a prodrug of butyric acid, resulted in AN-7-treated tumors being uniformly positive for PSA -indicative of differentiation [28]. Martirosyan and colleagues showed that five quinoline compounds based compounds inhibited HDAC activity *in vitro and* stimulated cell differentiation at growth inhibitory concentrations in MCF-7 breast carcinoma cells *in vitro* [29]. Munster and colleagues showed that treatment with SAHA (suberoylanilide hydroxamic acid or vorinostat), resulted in significant changes in the morphology of MCF-7 breast carcinoma cells suggestive of epithelial mammary differentiation [30]. Sakimura and colleagues [31] observed that intraperitoneal administration of depsipeptide to chondrosarcomas xenografted in nude mice resulted in down-regulated the synthesis of glycosaminoglycans and an elevation of alkaline phosphatase activity; both consistent with chondrosarcoma differentiation.

## Retinoids

Retinoids are a class of compounds derived from vitamin A that have demonstrated the ability to regulate cell proliferation, differentiation and apoptosis in normal and cancer cells [32]. Retinoids are believed to exert their effects by binding to retinoic acid receptors (RARs) that exist as 3 isoforms (RAR-α/- β /-γ).

Treatment of osteosarcoma and chondrosarcoma cell lines with ATRA have resulted in reversible growth inhibition and a decrease in colony formation [33, 34]. Further *in vitro* studies have showed that retinoic acid treatment results in hypophosphorylation of RARα inhibiting cellular proliferation and inducing osteoblastic differentiation which may provide a potential mechanism to explain the clinical effects observed [35]. A similar anti-proliferation and pro-differentiation effect has also been described for rhabdomyosarcoma (RMS) treated with retinoids. Use of retinoids in a variety of RMS cell lines derived from either alveolar or embryonal RMS displayed a reduction of cell proliferation along with a concomitant induction of myogenic differentiation [36-39]. Knockdown of XAB2, believed to be a component of the RAR corepressor complex, is able to increase ATRA-induced differentiation in RMS, APML and an ATRA-resistant neuroblastoma cell lines. Xenografted cells did exhibit a reduction of proliferation and morphologic evidence of muscle differentiation following ATRA treatment *in vitro*. Levels of the cyclin-dependent kinase inhibitors (CDKIs) p18, p21 and p27 were shown to be increased in all treated RMS cell lines but no CDK4 inhibition or hypophosphorylation of the Rb protein suggesting that ATRA-induced differentiation was not sufficient to induce cell cycle arrest in RMS cells. However, it is important to note that although ATRA did not reduce the time to relapse, the tumors obtained from ATRA-treated mice showed evidence of enhanced muscle differentiation (increased expression of the terminal muscle differentiation marker myosin heavy chain). Despite the variable outcomes following retinoid treatment of sarcoma cells, there continues to be a significant body of research to support further investigations into the anti-tumorigenic effects of retinoid-based differentiation of sarcomas, particularly for osteosarcoma and rhabdomyosarcoma.

## PPARγ Agonists

Peroxisome proliferator-activated receptor - γ (PPARγ) has been shown to be an important regulator of cell proliferation, differentiation and apoptosis in a variety of cell types including hepatocytes, fibroblasts, myoblasts and adipocytes [40, 41]. The biological effects of PPARγ have been found to be related to cell type and the specific ligand binding to PPARγ. Endogenous ligands of PPARγ include several unsaturated fatty acids and metabolites of arachidonic acid such as 15-deoxy-Δ 12,14-prostagladin J_2_ (15d-PGJ_2_). Synthetic ligands of PPARγ have been developed and include the thiazolidinediones (e.g. troglitazone, rosiglitazone), oral agents used in the management of type 2 diabetes mellitus, and non-steroidal anti-inflammatory drugs (NSAIDs; e.g. indomethacin).

Morrison and Farmer demonstrated that treatment of 3T3-L1 preadipocytes and murine fibroblast cell lines with the PPARγ agonist troglitazone resulted in the induction of the cyclin-dependent kinase inhibitors (CDKIs) p18 and p21 allowing for withdrawal of cells from the cell cycle and initiation of terminal adipogenic differentiation [42]. Development of knockout mouse models of PPARγ have revealed its importance in the development of adipose, placental and cardiac tissue as the null-PPARγ phenotype is embryonic lethal [43]. Activation of PPARγ with either endogenous PPARγ agonists (e.g. 15d-PGJ_2_) or synthetic agonists (e.g. RSG) have been effective in inducing cell cycle exit followed by terminal differentiation of preadipocytes and fibroblast cells implicating PPARγ activation in the regulation of lipid homeostasis [42, 44, 45].

Cellular differentiation has also been observed in cancer cell lines. Elstner and colleagues treated the breast cancer cell line MCF7 with the PPARγ agonist troglitazone (TGZ) and observed the inhibition of proliferation along with lipid accumulation. The combination of TGZ with the RXR agonist, ATRA, had a synergistic effect in irreversibly inhibiting MCF7 growth, reduction of BCL2 expression, and apoptosis of cells [46]. Relatedly, Sarraf and colleagues treated colon carcinoma cells with TGZ and demonstrated an increase in the levels of carcinoembryonic antigen (CEA), a marker of differentiation in colon carcinoma cell lines [47]. Perhaps one of the more pronounced effects of PPARγ activation is seen with PPARγ agonist treatment of liposarcoma. Tontonoz and colleagues were able to show that primary human liposarcoma (LPS) cells were effectively induced to undergo terminal adipocytic differentiation following treatment with the PPARγ agonist, pioglitazone [48]. Furthermore, they demonstrated additive effects in inducing adipocytic differentiation when LPS cells were treated with a combination of pioglitazone and an RXRα-specific ligand, LG268. These promising preclinical results regarding the differentiative effects of PPARγ agonist treatment in liposarcoma have been subsequently pursued in a clinical phase II trial utilizing the PPARγ agonist rosiglitazone [49].

## Trabectedin

Trabectedin (ecteinascidin-743 or ET-743; Yondelis) is a compound derived from the Caribbean tunicate *Ecteinascidia turbinata* and was discovered to have anti-tumor activity in the early 1970s through a survey of pharmacologic activity in plant- and marine-derived materials sponsored by the National Cancer Institute [50]. Binding of trabectedin to the DNA minor groove at the N2 position of guanine is believed to result in conformational changes of the DNA double helix disrupting the binding of transcription factors and potentially underlying its anti-tumor effects in select cancers [51-54]. Forni and colleagues evaluated the effects of trabectedin treatment in myxoid liposarcoma cell lines and found that trabectedin induces the dissociation of TLS-CHOP from target promoter sequences based on a series of chromatin immunoprecipitation experiments [55]. RT-PCR and Western immunoblot analyses of TLS-CHOP revealed stable expression levels suggesting that trabectedin does not result in the downregulation of TLS-CHOP expression. The dissociation of TLS-CHOP from target promoters results in the downregulation of TLS-CHOP target genes (e.g. CHOP, PTX3, FN1), but consequently induces an adipogenic differentiation program involving re-expression of the CAAT/enhancer binding protein (C/EBP) transcription factor family of genes which play key roles in regulating adipogenic differentiation. In MLS, TLS-CHOP is believed to sequester C/EBPβ preventing binding to its promoter and blocking progression of the adipogenic differentiation program. Trabectedin disrupts the association of TLS-CHOP and C/EBPβ allowing C/EBPβ activation of C/EBPα resulting in the initiation of terminal adipogenic differentiation. The novel mechanism of action of trabectedin coupled with its demonstrated efficacy in preclinical models either singly or in combination with converntional chemotherapy drugs makes it a promising candidate for continued investigation in formal clinical trials of patients with soft tissue sarcoma.

## Differentiation-based therapy in patients

To this point it could be argued that the differentiation effects described have been limited to cell lines and/or observed primarily under *in vitro* conditions. However, we would contend that differentiation as an *in vivo* or even a clinical end point has never been primarily sought out and thus rarely examined. We now provide clinical examples to support the preclinical data cited above.

NCI6338 evaluated the HDACI depsipeptide in patients with progressive recurrent and/or metastatic non-medullary radioactive iodine (RAI)-refractory thyroid cancer. This trial also evaluated the ability of depsipeptide to promote differentiation via assessment of increased RAI avidity in these tumors. Importantly, in a proof of principle event, clinically observed differentiation of thyroid tumor cells was observed with significant restoration of RAI avidity in two patients [56].

Demetri and colleagues conducted a small clinical trial of the PPARγ agonist troglitazone (TGZ) in patients with liposarcoma [57]. Serial biopsy results on 3 patients treated with TGZ revealed histologic changes consistent with increased lipid accumulation in tumor biopsy samples as well as marked increases in tumor triglycerides and phosphatidylcholine levels, and decreases in Ki-67 cell proliferation marker expression. Interestingly, the degree of adipocytic differentiation based on tumor triglyceride levels induced by TGZ treatment was most pronounced in a patient with the myxoid/round cell LPS variant. Furthermore, increases in fat density signal based on serial MRI examination of one patient with pleomorphic LPS was observed. However, such responses could not be validated in a subsequent trial [49].

In our own clinical experience we have observed the potent and rapid adipogenic differentiation effects of trabectedin on one patient with a myxoid round cell liposarcoma (MRCLS) [54] confirming previous reports of such rare differentiation-based responses in this sarcoma sub-type to trabectedin treatment [58]. As we have recently reported, a heavily pre-treated and locally recurrent large MRCLS (i.e., tumor displacement of the diaphragm and liver with complete extention into the pelvis) underwent a significant change in density from soft-tissue to fat corresponding to a 10 kg weight loss following several cycles of single-agent trabectedin. The patient also exhibited significant clinical improvement, but without radiological evidence of changes in tumor size measurements (i.e., stable disease as defined by RECIST criteria) [54]. The patient, whose disease was rapidly progressing at the time of trabectedin treatment, lived another two years without the need for surgical resection. At the time of our publication [54], he had begun to progress with areas of high grade sarcoma reasserting itself in the areas of well-differentiated fat. However, we are happy to report that he recently underwent aggressive surgical resection which removed a 55 pound mostly well-differentiated tumor/fat mass. Thus, review of the available literature on the potential of trabectedin and PPARγ agonists coupled with case reports on the efficacy of these agents in solid tumors highlights the relevancy and future promise of differentiation-based therapy as a novel treatment strategy for sarcoma patients warranting further investigation.

## Elucidating the mechanisms behind rare clinical observations

It is easy to relegate the above examples of clinical solid tumor differentiation therapy as anectodes and/or “fascinomas” of clinical medicine. In our own experience only one of 7 patients with MRCLS responded in such a manner to trabectedin. One could then ask, “Why?” If trabectedin works (as described above) via the reversal of TLS-CHOP-mediated sequestration of pro-adipogenic transcription factors resulting in re-initiation of the adipogenic program, why did only one of 7 patients experience the differentiation effect? Our recent work identified that this patient had one specific sub-type of TLS-CHOP (type II) and that this was also the only patient who possessed a mutated form of p53 [54]. Knowing this, we were able to generate an MRCLS mouse model that not only recapitulated MRCLS histopathology, but also its differentiation-based response to trabectedin. Perhaps more importantly we were able to use this model to demonstrate two important points: (a) ET-743 (trabectedin) downregulates TLS-CHOP expression post-transcriptionally; and (b) once adipocytic differentiation has been re-initiated via the actions of trabectedin, PPARγ agonists can then accelerate the differentiation process.

We propose that once the biology of a cancer is better understood, differentiation therapy loses its mysticism. In short, as we, as a scientific-medical community, uncover both the developmental origins of cancer as well as the differentiation pathways that promote the normal maturation of cells, the re-institution of the latter (i.e., normal differentiation) into the former (i.e., cancer cells) will become more and more routine. As a result, we will undoubtedly look back at these days of one-fit-for-all cytotoxic chemotherapies as not only overly simplistic but literally, not just figuratively, off-target.

## Going forward with differentiation therapy

**Figure 1:**
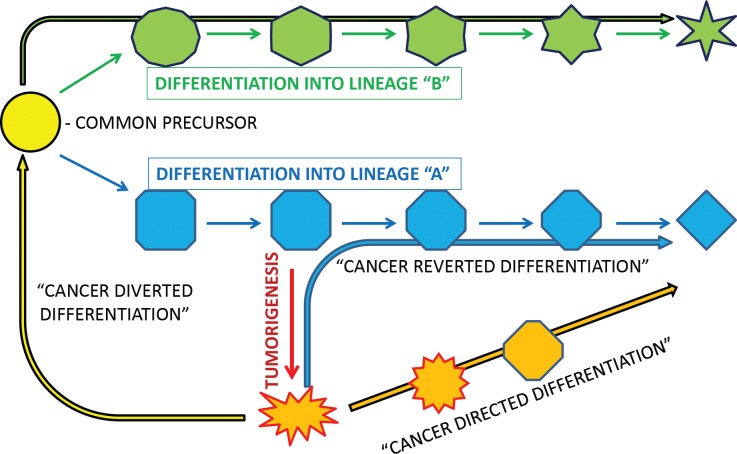
Schematic representation and suggested terminology for different ways in which cancer may be differentiatied

In this review, we have provided pre-clinical, clinical and mechanistic evidence for solid tumor differentiation therapy. But to imply that all differentiation therapy is going to be the same would be to simplify what is likely an extremely complicated phenomenon. We now present three ways in which cancer differentiation therapy can theoretically occur (Figure 1): 1) cancer directed differentiation; 2) cancer reverted differentiation; and 3) cancer diverted differentiation. In cancer directed differentiation, differentiation pathways are activated without correcting the underlying oncogenic mechanisms that have resulted in the initial differentiation block. In cancer reverted differentiation, correction of the underlying oncogenic mechanism results in natural restoration of endogenous differentiation pathways. Lastly, in cancer diverted differentiation, the cancer cell is redirected to an earlier stage of differentiation where access to alternative differentiation routes may be feasible. Thus, the cancer cell may then differentiate along an alternative lineage towards one in which its differentiation was not blocked.

Although it is tempting to ascribe the previously presented examples of differentiation therapy to the various mechanistic models of differentiation therapy just described, we will not do so here as such an assignment would imply that we completely understand the means in which differentiation is achieved in these cancer cells. Finally, we believe that there is great plasticity to these processes and differentiation therapy is likely to follow a spectrum of possibilities that may ultimately, at best, be only approximated by the models described above.

The general end point for cancer therapeutics has revolved around the idea of a complete eradication of cancer cells. Although adoption of a differentiation-based approach to this tenet of medical therapy may seem like a consolation, the potential for reversion of the malignant cancer phenotype to a more benign, or at the very least a lower grade of biologic aggressiveness, may serve as a critical clinical and biologic transition of a uniformly fatal cancer into one more amenable to management or to treatment using conventional therapeutic approaches. For differentiation therapy to be successful, it does not need to eliminate all the cancer cells or even differentiate them all to “normal” mature cells. If successful, the change in pathological status alone (e.g., from high grade to low grade or poorly to well differentiated), accomplished using differentiation therapy, will change the prognosis of most patients with cancer by decades.
